# Making Big Business Everybody’s Business: Aboriginal leaders’ perspectives on commercial activities influencing Aboriginal health in Victoria, Australia

**DOI:** 10.1186/s12992-024-01038-8

**Published:** 2024-04-18

**Authors:** Alessandro Connor Crocetti, Troy Walker, Fiona Mitchell, Simone Sherriff, Karen Hill, Yin Paradies, Kathryn Backholer, Jennifer Browne

**Affiliations:** 1 Deakin University, Institute for Health Transformation, Global Centre for Preventive Health and Nutrition, School of Health and Social Development, Faculty of Health, Geelong, VIC Australia; 2 Deakin Rural Health, Faculty of Health, Deakin University, Warrnambool, VIC Australia; 3 The Poche Centre for Indigenous Health, Faculty of Medicine and Health, University of Sydney, Sydney, Australia; 4 Alfred Deakin Institute for Citizenship and Globalisation, Deakin University, Burwood, VIC Australia

**Keywords:** Indigenous health, Commercial determinants of health, Health equity

## Abstract

**Background:**

The commercial determinants of health is a rapidly expanding field of research; however Indigenous perspectives remain notably underrepresented. For Indigenous peoples the intersection of globalisation, colonialism and capitalism may amplify commercially-driven health inequities. This study aimed to explore the perspectives of Aboriginal leaders regarding the influence of commercial activities on Aboriginal health and wellbeing in Victoria, Australia.

**Methods:**

Semi-structured interviews with 23 Aboriginal leaders from across five sectors (*n* = 15 urban, *n* = 8 rural/regional) were analysed through reflexive thematic analysis.

**Results:**

Three overarching themes were identified encompassing (i) harmful commercial practices and processes, (ii) improving corporate engagement and (iii) opportunities for self-determination through business. Participants expressed concern over aggressive marketing by the gambling industry, commercial exploitation of Aboriginal culture, the privatisation of public services, and lack of oversignt of corporate social responsibility strategies. Simultaneously, Aboriginal-led businesses were viewed as opportunities for cultural connection, and financial empowerment and self-determination.

**Conclusion:**

Numerous commercial entities and activities are perceived to influence Aboriginal health and wellbeing. This study highlights the need for stronger policy and regulation to mitigate harmful industry practices while incentivising the potential positive impacts of the commercial activities on Aboriginal health and wellbeing.

**Supplementary Information:**

The online version contains supplementary material available at 10.1186/s12992-024-01038-8.

## Background

Colonisation has had an enduring impact on Indigenous peoples’ health worldwide [1]. Contemporary expressions of settler colonialism include resource extraction, bureaucracy, capitalism, and an increasingly commercialised and commodified way of life [2]. Aboriginal and Torres Strait Islander peoples, the First Peoples of Australia, hold longstanding connections to the lands and waterways, alongside intricate knowledge systems spanning over sixty millennia [3–5]. These knowledge systems are underpinned by connection to culture, Country^1^, language, family, kinship and community, as well as self-determination [6, 7]. The physical, emotional, social, cultural and spiritual domains are interconnected in constructs of health for Indigenous peoples [8]. In recognition of this holistic conceptualisation, this study was guided by the National Aboriginal Community Controlled Health Organisation definition of Aboriginal health, which is *“not just the physical well-being of an individual but refers to the social, emotional and cultural well-being of the whole Community”* [9]

The importance of social factors in driving health disparities is well recognised [10]. It has been estimated that over one-third of the gap in health outcomes between Aboriginal and Torres Strait Islander and non-Indigenous people can be attributed to social determinants of health such as employment, income, housing, incarceration, racism and (in)access to services [11]. The link between racism and health outcomes is now well-established [12] and a 2011 survey of 755 Aboriginal peoples residing in Victoria found that 97% experienced racism in the last 12 months [13]. Evidence is also growing about the protective role of cultural determinants, including cultural expression, connection to Country, kinship and community, and self-determination in contributing to improved health and wellbeing for Aboriginal and Torres Strait Islander peoples [14–21]. Addressing the social and cultural determinants of health are central to national Aboriginal and Torres Strait Islander health policy [22]. 

Concurrently, the concept of commercial determinants of health (CDoH) has been increasingly recognised, both in Australia and internationally, as a public health priority [23, 24]. A recent Lancet series provided a comprehensive conceptualisation of commercial entities’ impact on health and a call to action for governments and the commercial sector to address the CDoH [25–27]. Although much of the CDoH research focuses on the “strategies and approaches used by the private sector to promote products and choices that are detrimental to health” (p.895, 28), we were interested in both the positive and negative impacts of commercial activity and therefore adopted the neutral definition by Gilmore et al. (2023): “the systems, practices and pathways through which commercial actors drive health and equity”. (p.2, 25) This neutral definition aligns with the recommendation from our advisory group, who advocated for a strength-based approach. CDoH involve a playbook of interrelated political, financial, scientific, marketing, supply chain, labour and reputation management practices employed by commercial actors to maximise profits [28]. For example, many industries aggressively market harmful products while employing reputation management strategies to portray themselves as ‘part of the solution’ [29, 30]. In the political domain, corporations often seek to obstruct or weaken public health regulation through lobbying, political donations and the ‘revolving door’ of individuals working across the private and public sectors [31–33]. 

The Australian state of Victoria, despite being one of the smallest in land area of all Australian jurisdictions, is the second most populated with an estimated 6.7 million people [34]. The approximately 78,000 Aboriginal and/or Torres Strait Islander peoples living in Victoria make up 1.2% of the total Victorian population [35]. Of that 1.2%, 94.2% identify as Aboriginal^2^ [36]. The lands now known as Victoria comprise many distinct Aboriginal nations, with at least 38 different languages [37]. There are 33 Aboriginal Community Controlled Health Organisations across Victoria, who deliver comprehensive, culturally safe health and social services tailored to the needs of their local communities [38]. Victoria is also home to over 400 registered Indigenous businesses, which, by definition, are at least 50% owned by Aboriginal and/or Torres Strait Islander peoples [39]. 

Despite the recent surge in CDoH research from Australia and Victorian leadership of the recent Lancet series [40], the CDoH have been largely absent from Aboriginal and Torres Strait Islander health research and policy. Our systematic review of the commercial determinants of Indigenous health identified practices common to the CDoH literature such as marketing, lobbying and corporate social responsibility strategies by unhealthy commodity industries [41]. However, we also identified unique industries (e.g. clothing and creative arts) and practices impacting Indigenous health and particularly cultural wellbeing through exploitation of Indigenous land and imagery [41, 42]. The increasing recognition of the nutritional value of Aboriginal foods has seen rapid expansion of the Australian “bushfood” industry, predominantly by non-Indigenous companies entering the market [43, 44]. We also found evidence to suggest that the commercial sector can have a positive impact on Indigenous wellbeing, particularly when businesses are led by Indigenous peoples [41, 42]. Internationally, Eisenkraft Klein and Shawanda (2023) builds on our systematic review by focusing on the loss of traditional ways of knowing, being and doing that have come from commercial industries activities of the alcohol, pharmaceutical, infant formula and, ultra-processed food and tobacco industry within Indigenous communities in Canada and the USA [45]. 

The authors of this paper support Aboriginal and Torres Strait Islander peoples’ fundamental right to self-determination and sovereignty, in alignment with the principles set forth in the United Nations’ Declaration of the Rights of Indigenous Peoples (UNDRIP) and The Uluru Statement from the Heart [46, 47]. The UNDRIP asserts that Indigenous peoples have the right to have a voice in the development of policies that affect them [46]. Yet no previous studies have explored the perspectives of Aboriginal peoples regarding the impact of commercial activities on the health and wellbeing of Aboriginal peoples. This knowledge is crucial for informing the design of equity-focussed strategies to mitigate harmful commercial activities and support health-promoting ones. Therefore, this study sought to explore the perspectives of Aboriginal leaders in Victoria regarding the influence of commercial activities on Aboriginal health and wellbeing. Specifically, we aimed to answer the following two research questions (i) how do Aboriginal leaders perceive the influence of commercial activities on Aboriginal health and wellbeing in Victoria? (ii) how do Aboriginal leaders perceive the potential for business and the private sector to have a positive effect on Aboriginal health and wellbeing?

## Methods

### Study design

We used a qualitative descriptive design employing semi-structured interviews to explore Aboriginal leaders’ perspectives on commercial activities and their impact on Aboriginal health [48]. The study aimed to privilege the voices and experiences Aboriginal peoples in Victoria and followed the CONSolIDated critERia (the CONSIDER statement) for strengthening reporting of health research involving Indigenous peoples [49]. This project was undertaken in partnership with three Aboriginal organisations: the Victorian Aboriginal Community Controlled Health Organisation (VACCHO), the peak Victorian Aboriginal health body, A2B Personnel, an Aboriginal-owned recruitment and labour hire company, and Clothing the Gaps an Aboriginal streetwear brand and social enterprise. The study was guided by a project advisory group which consisted of representatives from these three organisations, along with an Aboriginal representative from the funding body. The research team and the advisory group co-designed all participant facing-materials, including the plain language statement and interview questions, as well as the recruitment strategy. The findings were presented back to, and approved by, the advisory group prior to publication. The advisory group emphasised the importance of strength-based approaches and that our findings should be reflective of positive as well as negative impacts of commercial activity on Aboriginal health. The framing of the findings was adjusted to reflect this.

### Researcher positionality

Reflecting on positionality and cross-cultural dynamics is important in Indigenous health research as it elucidates settler privilege and helps to ensure research is conducted in a culturally safe and strength-based manner that respects and acknowledges the diversity of Indigenous peoples [50, 51]. This study was undertaken by a team of Aboriginal (TW, FM, SS, YP), Torres Strait Islander (KH) and non-Aboriginal scholars (AC, JB, KB) who worked with Aboriginal organisations to co-design a research approach that privileges the knowledges and voices of Aboriginal peoples and resisted colonial perspectives. In line with the principles of ethical research with Indigenous peoples [52], all interviews were undertaken by an Aboriginal researcher (TW, FM or SS) and a non-Aboriginal researcher (AC or JB) working as equal partners to practice cultural safety. This study was part of the doctoral research program undertaken by the first author (AC) that was supervised by a senior Aboriginal researcher (YP) and non-Aboriginal researchers with experience in Aboriginal health (JB) and CDoH research (KB).

### Participant selection and recruitment

We aimed to recruit 20–30 interview participants based on the timeframe of the study and previous public health research with Aboriginal and Torres Strait Islander populations [53, 54]. We purposively sampled Aboriginal leaders from across urban and regional Victoria. Given this is a new area of study and based on the recommendations from our advisory group, we decided to interview Aboriginal leaders as they would be best placed to provide detailed insight given their positions within the Victorian Aboriginal community. These included people in leadership positions within Aboriginal CommunityControlled Organisations (ACCOs), Aboriginal business owners and other Aboriginal peoples experienced in health promotion, Aboriginal affairs policy, research, and media advocacy. Potential participants were initially selected based on individuals or organisations identified in our media analysis [42] and those considered by the research team and the advisory group to be rich sources of information based on their professional experience. Potential interviewees were invited to participate via an email sent to their organisational email address. In total, 91 individuals were invited to participate in the study. One follow-up email was sent if a response to the invitation was not received within 2 weeks. Additional participants were identified via snowball sampling.

### Data collection

Semi-structured interviews were conducted between March-October 2023. Interviews were undertaken either face-to-face or over Zoom/Microsoft Teams and documented using a voice recorder. Participants were asked a series of questions about their views and experiences regarding the commercial activities affecting Aboriginal peoples in Victoria, encompassing both positive and negative impacts on health and wellbeing. See Supplementary File 1 for the interview guide. Interview recordings were transcribed by the first author (AC) and returned to participants so they had the opportunity to make edits. No participants made any changes to their transcripts.

### Data analysis

**Fig. 1:**
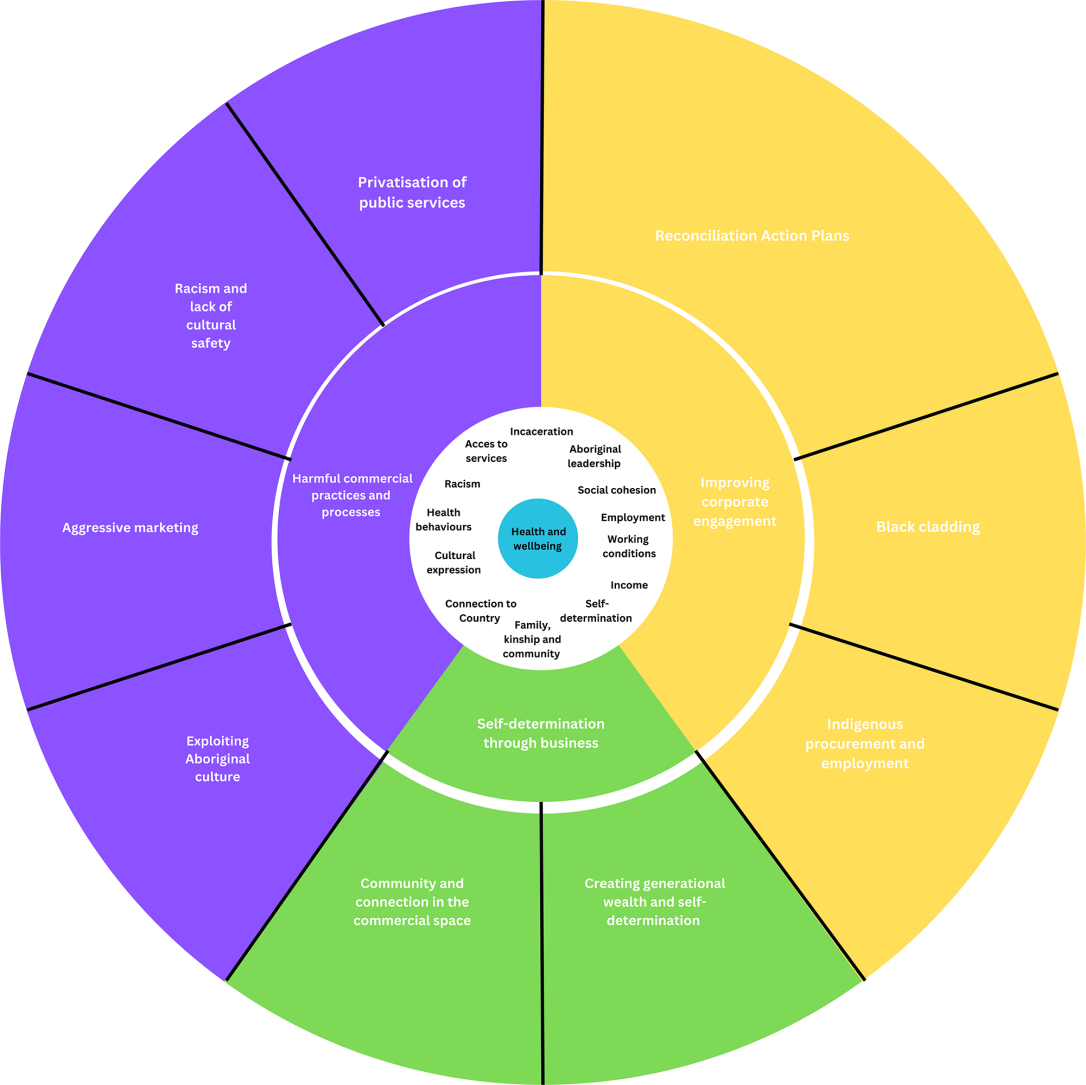
Concept map

Data analysis was guided by the reflexive thematic analysis method outlined by Braun and Clarke [55–58]. Coding was an inductive process informed by literature on the social and cultural determinants of Aboriginal and Torres Strait Islander health and the commercial determinants of health [6, 7, 25–27, 59]. AC performed preliminary coding in Microsoft Word (2016), based on the research questions. The evolution of codes was tracked using a Microsoft Excel spreadsheet, highlighting the iterations across each successive column. Initial codes were discussed with one Aboriginal (TW) and one non-Aboriginal (JB) member of the research team to create a coding framework. Similar codes were then grouped together to form themes. Theme names were reviewed and further refined in consultation with the research team. Throughout the data analysis process, Indigenous and non-Indigenous members of the team met for weekly culturally safe reflexivity discussions (a process we have embedded in our research team) to reflect on our positionality in the research process and how this may influence our practice and the research findings. Once the research team agreed on the themes, a concept map (Fig. 1) was created to visually represent and conceptualise the findings.

### Ethics

This study was conducted according to the guidelines for Ethical Conduct in Aboriginal and Torres Strait Islander Health Research [60] and approved by the Deakin University Human Research Ethics Committee, who consult with Aboriginal reviewers on research projects involving Aboriginal participants (approval number 2022 − 305). Written informed consent was obtained from all interview participants. Participants received a gift card for an Aboriginal business of their choosing worth $AUD50 to reimburse them for their time and contribution to the project.

## Results

Of the 91 individuals who were invited to participate, 23 agreed. The main reason for non-participation was time constraints. Interview duration ranged from 31 to 98 min. Participants worked in Aboriginal Community Controlled Organisations (ACCO) (*n* = 8), government agencies (*n* = 2), Aboriginal businesses (*n* = 9), research organisations (*n* = 3) and in the media (*n* = 2). 58% (*n* = 14) of participants were female and the majority (*n* = 16) were based in urban areas of Victoria, with fewer (*n* = 8) in rural/regional areas. All participants were over 18 and identified as Aboriginal.

Three themes and nine sub-themes were constructed from the data. Figure 1 depicts how commercial activities influence Aboriginal health and wellbeing (at the centre) through health behaviours and determinants (the innermost ring), according to the Aboriginal leaders we interviewed. The outer-most ring represents the nine sub-themes and how they relate to the three overarching themes: Harmful commercial practices and processes, improving corporate engagement and self-determination through business. In the following sections, we describe each theme and sub-theme, along with illustrative quotes from participants. Each quote is attributed with the participant’s code and sector.

Figure 1 illustrates a concept map of the commercial activities influencing Aboriginal health and wellbeing. Aboriginal health and wellbeing is at the centre, intertwined with health behaviours and determinants (the innermost ring). While, the outer-most ring represents the nine sub-themes and how they relate to the three-overarching themes: Harmful commercial practices and processes, improving corporate engagement and self-determination through business.

### Harmful commercial practices and processes

Participants described the harmful effects of the activities of various commodity industries. The ultra-processed food, infant formula, tobacco/vape, alcohol, mining, pharmaceutical and gambling industries were all discussed in relation to Aboriginal health, as well as the exploitation of Aboriginal cultural knowledge by non-Aboriginal businesses. Table 1 lists the number of participants that mentioned each industry. For example, several participants mentioned *“mining companies that destroy Country and the detriment that is playing to Mob’s*^3^*health”* (P11 Research). While another participant expressed concern about targeted marketing from the infant formula industry through *“the mining of pregnancy apps for information to target our communities”* (P8 ACCO). Although participants acknowledged that businesses fundamentally exist to make money, several questioned the ethics of companies that *“make a profit from the poorest people in the country*” (P19 Research). The harmful commercial practices and processes discussed by participants were grouped into four subthemes: aggressive marketing, exploiting Aboriginal culture, privatisation of public services and racism and lack of cultural safety. These are elaborated below.

### Aggressive marketing

The health harming impact of aggressive marketing of unhealthy, often addictive, products was emphasised by many participants. Marketing was described as “*a very strategic tool to prey on the people that struggle the most*” (P5 Business) and a technique to “*hook ya in”* (P4 Business). Participants were particularly concerned about gambling marketing, with some respondents noting they *“get a lot of gambling ads”* in their social media feeds (P15 Government). The relationship between gambling and sport was raised by several participants, noting that the marketing often appealed to children and could attract younger generations into gambling. One participant reflected *“you see it everywhere, my kids are starting to say ‘they got good odds’”* (P11 Research). Another participant shared a story about.

their four-year old son’s first experience at a football game at the Melbourne Cricket Ground (MCG):

***We took two steps out of the MCG and his first words were “I can’t wait to start gambling.” He was brand new to this ad campaign[…] I know the impacts, I know what it’s going to do for families, for people that can’t control it once they’re addicted[…] because they had made gambling look like fun.*(P12 Media)**.

**Table 1:** Number of participants discussing each industry

Industry	No of participants
Aboriginal business	13
Gambling	11
Art	9
Food and beverage	8
Alcohol	6
Media (including social media)	6
Private prison	6
Bushfood	5
Mining	5
Tobacco and e-cigarettes	5
Consultancy	4
Infant formula	3
Clothing	2
Dietary supplement	2
Pharmaceutical	2
Private healthcare	2
Sport	2

Most participants highlighted the need for stronger regulations to mitigate the marketing of the gambling industry. This was particularly the case for sports betting companies, which participants described as *“immoral”* (P10 ACCO) and likely to “*prey on the vulnerable*” (P4 Business) and the fact that *“Black fellas*^4^*love their sports”* (P13 Business). Participants from the Aboriginal Community Controlled sector noted that insufficient resourcing had been directed towards the prevention of gambling-related harms compared to other addictive products:

***I reckon it has a humongous impact on our Mob - governments look at drug and alcohol issues, gambling seems to be the poor cousin, it doesn’t get the attention that it needs and yet it does just the same amount of damage*(P10 ACCO)**.

### Exploiting Aboriginal culture

For several participants, exploitation of Aboriginal art and traditional foods was highlighted as a specific way in which commercial actors negatively impact Aboriginal people’s health and wellbeing. Participants underscored the need to protect artists’ Indigenous Cultural and Intellectual Property (ICIP) from commercial exploitation or as one participant described it: “*the bastardising of our cultural IP*” (P11 Research). Other participants described the unethical practices of businesses who sold fake Aboriginal art or underpaid Aboriginal artists for their work as *“degrading and [it] hurts the soul”* (P13 Business). A prominent example given by several participants was the attempted commercialisation of the Aboriginal flag by non-Aboriginal companies following a licencing arrangement with its artist. One participant reflected on the health and well-being impact of this:

***We saw this invisibility cloak come around the Aboriginal flag […] and it was really upsetting to see that because it was a symbol that united people and then all of a sudden it felt like it got taken away.*(P6 Business)**.

Several participants also raised the issue that most companies selling traditional Aboriginal foods are not owned and led by Aboriginal people and that certain “bushfood” companies reportedly represent themselves as Aboriginal businesses to appear more appealing to consumers. One business owner describes non-Indigenous bushfood companies as *“tokenistic, it pulls on the heartstrings of allies and Mob alike”* and that these companies need to have *“a lot more transparency”.* (P18 Business). Ironically, while non-Indigenous companies are attempting to capitalise on the reported health benefits of Aboriginal foods, the fact “less than 2% of the native food industry is run by Aboriginal people” (P15 Government) suggests that it is non-Indigenous businesses, rather than Aboriginal people, that are benefitting from Aboriginal cultural knowledge.

### Privatisation of public services

Privatisation of essential services was a major concern for some participants, with particular focus on the prison system. Participants emphasised the overrepresentation of Aboriginal peoples in prisons and the outsourcing of prison health services to private companies. One participant from the ACCO sector highlighted the consequences, stating *“we’ve got a lot of deaths in custody because they’re driven by the bottom line, that profit, not by the wellbeing outcomes or human rights”* (P10 ACCO). The health impact of privatisation was reinforced by another participant:

***We’ve seen a number of cases in private prisons where they’ve not provided adequate medical service and there have been dreadful consequences. I’m personally opposed to the privatisation of prisons, it’s a State responsiblity*(P19 Research)**.

A related concern raised by many participants was the perceived shift in government agencies and services towards a operating “*like a business model”* (P8 ACCO). Likewise, there was unease expressed about the increasing allocation of public money, including in the Aboriginal health portfolio, to private consultancy firms contracted to provide policy advice. These contracting practices were criticised as *“unacceptable distortions of our governmental system”* that ultimately yields *“second rate advice”* (P19 Research). Other participants shared the view that the system of outsourcing public service work incentivised the consultancy industry to “*set up as a revenue stream to get access to Indigenous allocations,”* (P22 Media) with concerns that *“everyone wants to get a piece of the pie and the Aboriginal voice is getting lost”* (P16 Business). Outsourcing Aboriginal health projects to private companies was also described as *“taking away the workforce from ACCOs because they’re paying better”* (P9 ACCO). According to one participant’s perspective, private entities pursuing profits from Aboriginal communities seemed to regard Aboriginal peoples as “*just this lemon that never runs out of juice that they can keep squeezing and squeezing”* (P22 Media).

### Racism and lack of cultural safety

Participants consistently discussed the pervasiveness of racism within commercial spaces, and its impact on daily life, including shopping and the workplace. For example, one participant recounted how Aboriginal people often had to think twice before entering a business owned by non-Aboriginal people due to concerns about discrimination *“because you’re not sure how you’re gonna get treated”* (P4 Business). Commercial entities were described as perpetuating a narrative focussed on Aboriginal disadvantage, which was perceived as demoralising.

***They saturate it from a deficit discourse right from the beginning, it’s all about deficit, it’s all about the higher risks and closing the gap…it’s in every organisation that I’ve either worked in or been a part of*(P7 Government).**

Many participants highlighted the lack of cultural safety within private sector workplaces. As one participant noted, *“the challenge that businesses have is not truly understanding the Aboriginal culture”* (P16 Business). Participants voiced concerns about the superficial nature of cultural safety training programs offered in workplaces which ranged from *“nothing at all”* (P5 Business) to “*a tiny little flick through an online learning thing that’s not very in-depth”* (P15 Government). Another participant stressed the importance of actively engaging with Aboriginal staff to understand *“what it is that they need to feel supported within a workplace”* (P9 ACCO), emphasising that tokenistic gestures such as *“putting Aboriginal art on the wall”* (P9 ACCO) fell short of addressing the needs of Aboriginal employees. Underscoring the need for cultural transformation within mainstream workplaces, one participant shared their experience of being recruited to a dedicated Aboriginal position and feeling as though they were merely “*touted around as ‘here look, how great are we, we have an Aboriginal staff member’”* (P15 Government). Some participants called for increased representation of Aboriginal peoples in senior positions within the private sector to reduce the structural barriers to employment for Aboriginal peoples.

***They need them in senior positions. It’s not good enough anymore to say that Aboriginal people aren’t qualified. It’s simply not true, it’s become a racist mantra, hasn’t it?*(P19 Research)**.

### Improving corporate engagement

Participants shared their insights into the dynamics of corporate sector engagement with Aboriginal communities, including the potential benefits and shortcomings of Reconciliation Action Plans and Indigenous employment and procurement policies. Many emphasised the tokenistic and transactional nature of these interactions, expressing the view that Aboriginal peoples felt like *“an add on”* in various industries (P5 Business) and that *“when commercial businesses are engaging with First Nations communities it’s never a service, it’s always a transaction”* (P22 Media). Some Aboriginal health organisations, acutely aware of the the transactional nature of commercial partnerships, reported strategically maximising the benefits for their health programs, as expressed in the following quote:

**The purpose of the partnerships is one for them so they can tick their boxes because they’ve got to increase their community engagement with Indigenous communities so let’s be real about that. But for us it’s like alright I’ll scratch your back if you scratch my back […] and it’s a non-financial partnership” (P17 ACCO)**.

### Reconciliation Action Plans

Reconciliation Action Plans (RAPs) are voluntary strategic frameworks that list a company or organisation’s desire to build relationships with Aboriginal and Torres Strait Islander peoples [61]. Participants’ views on the effectiveness of RAPs were mixed. Some spoke about RAPs in a positive light, suggesting that they are *“fantastic for businesses and corporations”* (P12 Media) *and “a force for good in overcoming the disadvantages in closing the gaps”* (P19 Research). While other participants questioned whether they were genuinely impactful or merely symbolic with comments such as *“I don’t think they’re effective at all”* (P9 ACCO). Another participant reflected that they *“have seen some good ones but have a higher stack of really obsolete ones”* (P7 Government). Critics argued that RAPs could be used by companies to *“blackwash their performance”* (P19 Research), suggesting they were often tokenistic, did not empower Aboriginal peoples and reinforced corporate colonial practices. As one participant explained, they are *“designed for the mainstream populations to keep taking up more space [and] feel good about themselves”* (P22 Media). This perspective was reinforced by this Aboriginal health leader’s statements on the process and design of RAPs:

**Every RAP plan is built the same way– get an artwork make it look deadly, we’ll do a launch program, we’ll build it around how many people get employment and then we’ll do it for three years and we’ll engage three or four Black people around the area, and we’ll just allow that to be the narrative (P5 ACCO)**.

Concerns were raised about the lack of clear accountability mechanisms in the RAP process. Participants asked *“who evaluates what differences RAPs have made?”* (P10 ACCO) and “*does anybody actually read them?”* (P13 Business). Some participants suggested that the burden was often placed on Aboriginal staff to shape and uphold the principles of the RAP which *“can be really draining for Aboriginal people”* (P14 ACCO). Others called for companies to demonstrate leadership by thoroughly examining their strategic plans and take concrete action to address structural issues affecting Aboriginal peoples.

### Indigenous procurement and employment

Participants described Indigenous employment and procurement practices, including the impact of the Victorian government’s social procurement framework on Aboriginal businesses and employees. Participants expressed mixed perspectives about the effectiveness of these initiatives. Some participants highlighted the benefits of the social procurement framework, which directs government personnel to prioritise purchasing from social enterprises or Aboriginal-owned businesses, as it enables Aboriginal businesses to secure “*a bigger cut of government expenditure*” (P15 Government). Similarly, several participants noted the increased Aboriginal workforce presence in the construction industry, as a result of government contracts stipulating “*it’s gotta be a percentage of your workforce as First Nations Australians*” (P17 ACCO). However, other participants raised concerns about corporate “*box ticking”* (P20, Business) to meet Aboriginal employment quotas, as well as a lack of wellbeing support for Aboriginal peoples. Some participants reported that this lack of support could perpetuate negative stereotypes or cause “*more trauma, more violence, more pain, it’s just destroyed people under the guise of progress”* (P22 Media).

### Black cladding

The issue of “black cladding”, where non-Indigenous businesses exploit Indigenous identity for commercial gain [62] was raised by several participants. One participant described this practice as “*businesses that aren’t really a Black business but are putting Black fellas at the front”* (P10 ACCO) in order to win government contracts. Participants expressed concerns about the impact that black cladding has on Aboriginal wellbeing as it “*creates further fraction”* and exacerbates frustration within the Aboriginal community (P16 Business). They noted that non-Indigenous businesses sometimes used ambiguous language to market themselves, implying they supported Aboriginal communities to reap social and economic benefits, as this participant explained:

***They say things like “we respect the local Aboriginal community”. Like, do you respect them? Do you do anything active to respect them? […] I think at the end of the day they’re businesses trying to sell product and having that kind of connection benefits their business but whether or not it’s genuine […] I think overall the dominant answer is no, but it’s that marketing (P15 Government)***.

### Self-determination through business

Self-determination was a key benefit of business ownership expressed by participants. The ability to operate independently of government funding which was often *“constrained to the White frameworks”* (P22 Media) was viewed as particularly empowering because *“if we own it, we can actually change more”* (P5 Business). Participants also highlighted how business and entrepreneurship provided employment opportunities for Aboriginal peoples beyond the traditional roles for Aboriginal staff in government-funded organisations. The following quote from an Aboriginal business owner encapsulates this sense of freedom and empowerment.

***I’ve never really experienced self-determination like I have in business. It’s because we’re not government-funded […] Essentially, it means that we have free choice on who we work with and what we choose to do, and it’s not influenced by anyone […] we have a whole lot of self-determination on how we choose to spend our profits, and how we choose to use our voice, and how we choose to use our platform (P6 Business)***.

Within this overarching theme of self-determination through business, there were two distinct subthemes related to the potential positive effects of employment in the private sector and Aboriginal business on health and wellbeing. These were *creating generational wealth and self-determination* and *community connection in the commercial space*.

### Creating generational wealth and self-determination

Many participants emphasised the potential of business to empower Aboriginal peoples in creating generational wealth and financial autonomy. Participants described working in the private sector as an opportunity to “*change the financial hardship that’s been the constant narrative”* (P5 Business) but also stressed that *“it’s not just about making money, it’s about being able to feel valued”* (P4 Business). However, some participants pointed out that individuals who are not accustomed to earning large amounts of money may require additional support when commencing work. Some suggested *“financial literacy should be taught”* (P4 Business) by corporations in a culturally safe and empowering manner, as explained by the following participant:

***Okay [if] Mob are going to earn six figures working in this industry, we’ve got to make sure that we’re putting in the resources ourselves [so] that it’s going to be a long-term growth for this individual and the ripple effect is going to impact this individual and their family (P17 ACCO)***.

All participants expressed enthusiastic support for Aboriginal businesses. Participants expressed a sense of pride that *“young ones are starting their own businesses”* (P1 ACCO), seeing Aboriginal designs in major retail stores and Aboriginal peoples leading the way in the business sector. Others emphasised that Aboriginal peoples should have the same opportunity to pursue careers in “*the commercial space”* because *“we’ve been locked out of it for years”*, (P11 Research). One Aboriginal business owner highlighted the transformative power in finding meaningful work and independence:

***It’s finding purpose, the more employment the more breaking away from the system, or that dependency or that victim mentality that we need the government to save us, (P16 Business)***

### Community and connection in the commercial space

Creating an Aboriginal presence and fostering solidarity in the commercial space was described by participants as an expression of cultural identity that had potential to “*change the system”* (P4 Business). Participants highlighted the importance of maintaining their cultural integrity in the corporate world, emphasising *“we shouldn’t have to dissolve our culture[…]to get the same opportunities”* (P16 Business). Many expressed appreciation for the values and ethos upheld by Aboriginal businesses, noting *“integrity is everything*” (P6 Business). Participants also emphasised that Aboriginal businesses are significantly more likely to employ Aboriginal peoples as they have a “*genuine intent to positively contribute to Community” (P15 Government).* This deeply ingrained sense of community and connection is uncommon in mainstream profit-focused commercial employers, as one business owner explained:

***[For commercial entities] it doesn’t really matter who you personally are outside of work. That’s irrelevant because that doesn’t affect the capital system but for First Nations people it does matter, we need to build rapport outside of work (P16 Business)***.

A critical aspect for many Aboriginal businesses is supporting the wellbeing of their staff alongside economic advancement as *“they treat you like family” (P2 Business).* As one participant explained, *“the benefits to our Mob around pastoral care, they factor in matters and things that are important to us like cultural leave”* (P11 Research). Participants emphasised that this wellbeing focus had a positive effect on Aboriginal employees and “*changed their lives for the better from a physical and mental health point of view*” (P20 Business).

## Discussion

To the best of our knowledge, this the first study exploring the perspectives of Indigenous peoples on the influence of commercial activities on health. Our interviews with Aboriginal leaders from diverse sectors and communities across Victoria build on our previous research in Australia and the work of Eisenkraft Klein and Shawanda in Canada, by shedding light on the positive and negative effects of contemporary commercial practices on health and its determinants for Aboriginal peoples in Victoria [41, 42, 63]. Three overarching findings can be drawn from this study. Firstly, our interviews uncovered the breadth of commercial practices adversely affecting Aboriginal communities, including aggressive marketing of harmful products, exploitation of Aboriginal cultural and intellectual property, privatisation of public services and racism in the private sector. Secondly, policies and plans aimed at improving commercial engagement with Aboriginal communities, including government social procurement policies and corporate social responsibility strategies, such as RAPs, require better oversight, monitoring, and evaluation. Lastly, business, particularly Aboriginal-owned businesses, can be an important source of employment, economic empowerment and self-determination for Aboriginal peoples.

The findings from the present study add to existing international evidence on the CDoH. Our concept map (Fig. 1) provides an initial, empirically-derived conceptualisation of the commercial activities influencing Aboriginal health and wellbeing. Current conceptualisations of CDoH describe the ways in which commercial activities influence health behaviours, exposures and practices through structural and environmental drivers such as work, school and living conditions [25]. Our findings extend this conceptualisation to illustrate how commercial activities influence the social and cultural determinants of health for Aboriginal peoples, such as racism, incarceration, family, kinship and community and cultural expression. Existing models of the CDoH also emphasise the political and economic systems and pathways through which commercial actors influence health [25]. Many of the commercial sector activities we identified intersect with public sector policies (e.g. procurement and contracting), echoing calls for a nuanced understanding of commercial entities in CDoH research [26]. 

Within the commercial determinants of health literature, there has been mounting research on the practices of unhealthy commodity industries, particularly the tobacco, alcohol, ultra-processed food, breast milk substitutes and sugar-sweetened beverage industries who use the same ‘playbook’ of practices to promote and sell their products [25, 28, 64]. Gambling marketing was a key concern raised by participants in our study, who suggested that gambling received less attention in Aboriginal health compared to other addiction issues. Australia has the highest per capita gambling losses in the world [65], with the social costs estimated to be $AUD7 billion in Victoria [66]. Aboriginal communities are disproportionately impacted by gambling harms [67]. The limited data available suggests that gambling adversely affects up to 20% of Aboriginal peoples compared to 3.2% of non-Indigenous Australians [67, 68]. With increasing concerns about the normalisation of gambling and the pervasive nature of gambling marketing in Australia, the findings of this study reinforce the need for stronger regulatory controls on the marketing of gambling in Australia and more rigorous monitoring of gambling prevalence and harms, including among Aboriginal peoples [65, 69]. 

The exploitation of Indigenous Cultural and Intellectual Property (ICIP) and the associated harms to cultural wellbeing was a unique commercial practices influencing Indigenous wellbeing identified by participants in our study. ICIP pertains to the rights of Indigenous peoples to their cultural heritage, including objects, sites, language, Indigenous knowledge and its expression [70]. Indigenous ecological knowledge is a fundamental aspect of ICIP encompassing knowledge of Country as well as native plants and animals [71]. A 2022 Australian Government Productivity Commission study on inauthentic Aboriginal and Torres Strait Islander art highlighted that inauthentic art is pervasive and causes cultural and economic harm to Aboriginal communities, noting that 75% of Aboriginal-style art is produced by non-Indigenous people [72]. Previous research on the significance of ICIP in Aboriginal health highlighted the emotional and cultural distress experienced by Aboriginal community members who were unable to use the Aboriginal flag due to licencing arrangements with non-Indigenous businesses [73]. The findings of the present study add to this evidence on the harm caused by commercial exploitation of Aboriginal culture including art and foods. Our findings align with international evidence documenting the tobacco industry’s appropriation of Indigenous imagery, symbols and culture to market and sell cigarettes and undermine tobacco control policies [74–76]. 

The rapid increase in privatisation and outsourcing of public services in Australia and its potential impact on health and social inequities has received limited attention in the CDoH literature [77, 78]. Privatisation is part of the political and economic system that enables commercial entities to cause harm [25]. Notably, Australia has the highest rate of private incarceration in the world and Aboriginal peoples are both overrepresented in the criminal justice system and significantly more likely to die while in prison [79–81]. Participants also raised concerns about the growing influence of consultancy firms advising governments on health and Aboriginal affairs policy, with potential conflicts of interest when these firms maintain both government and commercial clients [78, 82]. According to the Australian government’s procurement website, there were, as of October 2023, 1,578 active contracts between consultancy firms and federal government agencies responsible for Indigenous affairs [83], underscoring the extent to which the consultancy industry has embedded itself in the public sector. Over the past decade the ‘Big Four’ consultancy firms (Ernst & Young, PricewaterhouseCoopers, KPMG and Deloitte), have donated AUD4.2 million to the major Australian political parties, while their government contracts increased from AUD282 million to AUD1.4 billion from 2012 to 2022 [84]. 

Corporate social responsibility (CSR) involves the voluntary commitments of commercial entities to enhance ethical standards and promote societal wellbeing [85]. While this approach can have positive impacts, it is often perceived as a strategy to bolster a company’s reputation [25, 86]. The predominant example of CSR raised in this study was Reconciliation Action Plans (RAPs), which were viewed with mixed enthusiasm. Concerns were raised about corporate accountability and the burden placed on Aboriginal staff to educate non-Indigenous colleagues about the issues impacting Aboriginal and Torres Strait Islander communities [87]. Weenthunga Health Network, a Victorian Aboriginal health workforce organisation coined the term ‘colonial load’ [88], emphasising the cognitive load imposed by colonial instutions, racist and unsafe workplaces on Aboriginal staff [88]. Previous research has highlighted that CSR practices, including RAPs, and cultural awareness training are insufficient to mitigate the harms caused to Aboriginal communities by some commercial entities [89]. Although third-party CSR auditing organisations may enhance CSR effectiveness [90], RAP evaluations currently rely on limited surveys and infrequent self-reporting [91]. 

Employment is a key social determinant of health associated with numerous positive health outcomes [92], and Australian governments have committed to increasing Aboriginal and Torres Strait Islander employment through the Closing the Gap initiative [93]. Our findings support the literature that empowering Aboriginal communities through employment and business ownership promotes transformative change to financial wellbeing for communities who have faced generations of economic exclusion [94]. Our findings also underscore the ongoing challenges for Aboriginal employees within the mainstream private sector, including lack of cultural safety and workplace racism. Previous research has echoed these concerns, emphasising the inadequacy of cultural competency training, often viewed as mere “tick-the-box exercises”. (p.13, 95) Despite the growth in Aboriginal employment and income in recent years [95], workplaces remain one of the most common settings to experience racial discrimination which contributes to Aboriginal peoples being locked out of the economy [96]. 

Despite the numerous detrimental impacts of commercial activity identified in this study, a distinctive finding was the social, cultural, and economic benefits associated with Aboriginal-owned businesses, both for employees and business owners. Many of these insights align with prior research, which has consistently highlighted the higher levels of Aboriginal and Torres Strait Islander employment within Indigenous businesses compared to non-Indigenous businesses, the embedded cultural practices, and stronger connections with community networks [97–99]. The Victorian and federal governments have procurement policies with specific targets for awarding contracts to Aboriginal-owned businesses [100, 101]. Such policies have shown modest progress in Aboriginal employment within the construction sector, increasing from 8.9% in 2015 to 10% in 2021 [102]. However, participants in our study raised concerns about “Black-cladding”, where non-Indigenous businesses seek to exploit Aboriginal identity to win government contracts, potentially undermining the effectiveness of Aboriginal procurement policies. Currently, Victorian and federal governments define an Indigenous business as being at least 50% Aboriginal and/or Torres Strait Islander owned. To address concerns about Black cladding, Supply Nation, the national Indigenous business certifying body requires businesses to be majority (at least 51%) owned, managed and controlled by Aboriginal and/or Torres Strait Islander peoples [103]. The federal government is reviewing its definition of Indigenous business to inform the future of its Indigenous procurement policy [104]. 

### Strengths and limitations

This study is strengthened by the participation of Aboriginal peoples across various sectors and Aboriginal nations, including Aboriginal leaders in both metropolitan and rural/regional areas. Additionally, our methods and findings were enriched by the guidance of the project advisory committee and involvement of experienced Aboriginal researchers throughout the study. However, it is important to acknowledge the study’s limitations, particularly in terms of its geographic scope which was limited to Victoria. Although participants from a variety of sectors were interviewed, it was difficult to recruit politicians, senior public servants and Aboriginal people working in the corporate sector. It is also important to acknowledge that Aboriginal peoples may be employed within the industries discussed negatively by the interviewed participants, and their perspectives may be different. It is also possible that our interview guide influenced participants to discuss certain industries, however, the breadth of sectors discussed suggests this is unlikely. Our findings are based on the perspectives of 23 participants who may not represent the diversity of Aboriginal communities, cultures and experiences across Victoria or Australia. To address this gap, future CDoH research should include the voices Aboriginal and Torres Strait Islander peoples across the country, particularly in remote areas of Australia. Future research should build on our empirically-derived concept map to more completely conceptualise the systems, practices and pathways through which commercial actors drive health and wellbeing for Aboriginal and Torres Strait Islander peoples.

## Conclusions

While research on the CDoH is growing internationally, scarce attention has been paid to the ways in which commercial activity intersects with colonisation to influence health and its determinants for Indigenous peoples [45]. The present study has highlighted the perspectives of Aboriginal leaders about the ways in which commercial activities may be influencing health for Aboriginal peoples in Victoria. Despite the various commercial practices identified, the CDoH remain largely absent from Aboriginal and Torres Strait Islander health policies at both the state and federal levels [22, 93, 105]. In order to mitigate the harms caused to Aboriginal peoples by the commercial sector, both population-wide and targeted policy action is required. While enhanced regulation of the gambling, advertising and consultancy industries, as well as reforms to the prison system, would likely benefit all Australians, our findings suggest that these reforms, alongside greater regulation of the Aboriginal art and bushfoods industries, will also be of particular benefit for Aboriginal peoples. Finally, increasing support for Aboriginal-owned businesses, strengthening procurement policies and improved oversight of RAPs, will further promote Aboriginal employment, staff wellbeing and self-determination across the commercial sector.

### Electronic supplementary material

Below is the link to the electronic supplementary material.

Supplementary Material 1

## Data Availability

The qualitative interview data in this study are not publicly available.

## Footnotes

1 The Indigenous authors of this paper agree that the term, ‘Country’ extends beyond geography to signify a deep spiritual, cultural, and physical connection through their ancestral ties to the lands, sea, waterways, and skies. Therefore, ‘Country’ is recognised as a proper noun and is capitalised.

2 Aboriginal peoples are the Indigenous people of Victoria, therefore hereafter we will respectfully use the term Aboriginal when referring to Aboriginal and Torres Strait Islander peoples in Victoria.

3 The Indigenous authors of this paper use the term ‘mob’ to refer to a group of people. It is a culturally appropriate term often used to describe someone’s family, community, or people from the same cultural background. It is a term that reflects a sense of belonging and community ties.

4 The Indigenous authors of this paper agree that the term ‘Black fella’ is an Aboriginal English term typically used by Aboriginal and Torres Strait Islander peoples to refer to one another.
