# Supplementary material for: Making Big Business Everybody’s Business: Aboriginal leaders’ perspectives on commercial activities influencing Aboriginal health in Victoria, Australia
Source: Global Health. 2024 Apr 18;20:33. doi: 10.1186/s12992-024-01038-8 (PMC11025162; doi:10.1186/s12992-024-01038-8)
Supplement: Supplementary file 1 — Supplementary Material 1 [file 12992_2024_1038_MOESM1_ESM.docx]

| Making Big Business Everybody’s Business  Interview questions for Aboriginal participants in Victoria |
| --- |
| 1. Can you start by briefly telling us a bit about yourself. This could include your roles at work or in the community? |
| Today we would like to talk to you about the ways in which the activities of business or private companies influence Aboriginal health and well-being. This includes physical, social, emotional and cultural wellbeing.  When we talk about ‘activities’ of business or private companies, we’re talking about the things they may do on a day-to-day or even a one-off basis that may influence Aboriginal health, either positively or negatively. For example, this might relate to activities in the community or it might relate to the broader things companies do to sell their products or make more profits.   1. Can you tell us how you think the activities of business or private companies might influence Aboriginal health and if you have any examples you would like to share?   Prompt: Examples could include the tobacco/e-cigarette, alcohol, food, gambling or mining industries. [Keep prompting “Anything else?” until all relevant industries discussed]   1. What specific things are these industries or companies doing that may be influencing peoples’ health? Is there anything that stands out?  Prompt: E.g. supplying unhealthy products, advertising/marketing, unhealthy sports sponsorship, lobbying government, corporate social responsibility 2. How do these companies or activities impact Aboriginal health? or the way we eat? [If about food] 3. Can you provide any specific examples from your own experience? How’d this make you feel? 4. What do you think needs to happen to reduce any negative impacts these companies are having on Aboriginal health or wellbeing? Is there anything Government needs to do? |
| 1. Can you think of any businesses, industries or private companies that may be having a positive impact on Aboriginal health and wellbeing?   Prompt: Examples could include Aboriginal-owned businesses, Aboriginal tourism, Aboriginal fashion, Bush food. [Keep prompting “Anything else?” until all relevant industries discussed]   1. What specific things are these industries or companies doing that may be enhancing peoples’ health? Is there anything that stands out? 2. How do these activities companies/activities impact health and wellbeing? 3. Can you provide any specific examples from your own experience? How’d this make you feel? 4. How do you think we can encourage more businesses to do things like this? 5. Is there anything Government could do to support business to make a positive impact on health? |
| 4. Can you think of any examples of communities overcoming powerful companies to protect Aboriginal health or public health?   1. Have you or any other organisations you know been involved in any advocacy in response to private companies or big business? (If so can you tell us about this?) 2. What do you think are the key lessons for other communities about the most successful strategies against potentially harmful commercial activities? |
| 1. Given everything that we have discussed, is there anything else you would like to add before we finish up? Is there anyone else that you think we should speak to? |
